# Downregulation of p53 by Insufficient CTCF in CD4^+^ T Cells Is an Important Factor Inducing Acute Graft-Versus-Host Disease

**DOI:** 10.3389/fimmu.2020.568637

**Published:** 2020-09-29

**Authors:** Juan Hua, Yan Chen, Bin Fu, Xu Chen, Xue-jun Xu, Shuang-Hui Yang, Cong Chen, Ya-jing Xu

**Affiliations:** Department of Hematology, Xiangya Hospital, Central South University, Changsha, China

**Keywords:** aGVHD, CD4^+^ T cell, p53, CTCF, P300

## Abstract

Recent evidence indicates that p53 plays a protective role against various systemic autoimmune diseases by suppressing pro-inflammatory cytokine production and reducing the number of pathogenic T cells. However, whether abnormal p53 expression participates in the development of acute graft-versus-host disease (aGVHD) remains unclear. In this study, we demonstrated that p53 was downregulated in CD4^+^ T cells from patients with aGVHD compared with the non-aGVHD group. Furthermore, we confirmed that low expression of CCCTC-binding factor (CTCF) in CD4^+^ T cells from aGVHD cases is an important factor affecting histone H3K9/K14 hypoacetylation in the *p53* promoter and p53 downregulation. Restoring CTCF expression in CD4^+^ T cells from aGVHD patients increased p53 amounts and corrected the imbalance of Th17 cells/Tregs. Taken together, these results provide novel insights into p53 downregulation in CD4^+^ T cells from aGVHD patients.

## Introduction

Allogeneic hematopoietic stem cell transplantation (allo-HSCT) is considered the exclusive treatment option for hematopoietic malignancies, but acute graft-versus-host disease (aGVHD) is the primary limitation of this therapy. Despite prophylactic treatment with immunosuppressants, approximately 20–80% of allo-HSCT recipients develop aGVHD (1–3). The pathogenetic process of aGVHD involves a complex cascade of humoral and cellular interactions in which alloreactive donor T lymphocytes target host tissues, causing tissue injury through the secretion of proinflammatory cytokines and direct cytotoxicity (4–6). Therefore, controlling the overactivation of T lymphocytes is the key to preventing aGVHD. To achieve this goal, the pathogenesis of aGVHD needs further elucidation.

The transcription factor p53 is a tumor suppressor gene that plays an important role in regulating cell growth, apoptosis, and DNA repair. Mutations in the *p53* gene occur in half of all human malignant tumors (7–9). In recent years, evidence indicates that p53 plays a protective role in inflammatory response by inhibiting the inappropriate expression of pro-inflammatory factors such as IL-1, IL-6, IL-12, and TNF, etc. p53 dysfunction is related to the occurrence of inflammatory and autoimmune diseases (10–13). Recent studies demonstrated that inhibition of autoimmunity by p53 may be related to p53-mediated Foxp3 transcription in Tregs. Meanwhile, p53 activates Foxp3 transcription by directly binding to its promoter, which contributes to p53-mediated Treg induction (14, 15). In addition, p53 inhibits Th17 cell differentiation by suppressing the signal transducer and activator of transcription 3 (STAT3) and nuclear transcription factor-κB (NF-κB) pathways (14, 16). Thus, p53 plays an important role in regulating Th17 cell/Treg balance. Accumulating evidence demonstrates that the imbalance between Th17 cells and Tregs is an important cause of aGVHD (6, 17–19). The above findings clearly indicate that p53 is a key factor in inhibiting autoimmune and inflammatory responses; however, whether abnormal p53 expression participates in the development of aGVHD remains unclear.

Histone H3 acetylation at Lys 9 (H3K9ac), an important histone modification, is highly correlated with active promoter state (20). Histone H3 acetylation at Lys 14 (H3K14ac) shows a high co-occurrence with H3K9ac and is considered the hallmark of active gene promoters (21). In this study, we demonstrated that the CCCTC-binding factor (CTCF) in the p53 gene promoter that contributes to its transcriptional expression. Reduced CTCF leads to insufficient binding of E1A binding protein p300 (p300) in the p53 promoter region, which in turn causes H3K9/K14 hypoacetylation, and p53 transcription is repressed in CD4^+^ T cells from aGVHD patients. Taken together, these results provide novel insights into p53 downregulation in CD4^+^ T cells from aGVHD patients.

## Materials and Methods

### Subjects

A total of 90 patients administered allo-HSCT (2016–2019) from HLA-identical sibling donors at the Central of Hematopoietic Stem Cell Transplantation of Xiangya Hospital were included. This study was carried out in accordance with the recommendations of international ethical guidelines for biomedical research involving human subjects. The study protocol was approved by the Human Ethics Committee of Xiangya School of Medicine, Central South University. All subjects provided signed informed consent in accordance with the Declaration of Helsinki. The clinical characteristics of the patients are shown in Table 1. The median time from transplantation to aGVHD onset was 53 (20–91) days. Conditioning regimens were adopted as described in our previous study (22). Assessment of aGVHD was based on clinical symptoms in accordance with reported criteria (23, 24). The patients were divided into two groups, according to whether they had aGVHD. Peripheral blood samples from patients were collected as soon as aGVHD was diagnosed and before starting the therapy (*n* = 45). According to the onset time of aGVHD patients, we collected peripheral blood from three patients with the same disease type at the same time point after hematopoietic stem cell transplantation. Among these three patients, patients who had not developed aGVHD were included in the control group (*n* = 45). The transplant pattern was the same in both groups. In addition, the normal CD4^+^ T cells were obtained from three health medical staff recruited from Xiangya Hospital.

**TABLE 1:** Clinical characteristics of patients.

	No GVHD	aGVHD
Number	45	45
Median age	35	32
Sex (female/male)	20/25	19/26
**Diagnosis**		
ALL	17	16
AML	18	19
MDS	9	8
CML	1	2
**Acute GVHD grade**		
1		7
2		21
3		15
4		2
Days to aGVHD onset, median (range)		53 (range: 20–91)

### Isolation, Culture, and Transfection of CD4^+^ T Cells

CD4^+^ T cells were extracted from 60 ml venous peripheral blood with human CD4 beads (Miltenyi, Bergisch Gladbach, Germany) as directed by the manufacturer and cultured in RPMI 1640 (Thermo Fisher Scientific, MA, United States) supplemented with 10% fetal bovine serum (FBS) and 1% penicillin/streptomycin. The gene overexpression (pCMV6) and interference (pRS) plasmids were transfected into 2 × 10^6^ CD4^+^ T cells with Human T cell Nucleofector Kit and Amaxa Nucleofector (Lonza, Walkersville, MD, United States). Briefly, CD4^+^ T cells were isolated, resuspended in 100 μl human T cell Nucleofector solution, and mixed with the plasmid. The resulting mixture was electrotransfected with Nucleofector program V-024 on an Amaxa Nucleofector. The transfected cells were cultured in RPMI 1640 containing 10% FBS at 37°C with 5% CO_2_ and collected 48 h later.

### Proliferation of CD4^+^ T Cells

CD4^+^ T cells were cultured in 24-well plates (1 × 10^6^/ml), stimulated with recombinant human IL-2 (10 units/ml, eBioscience) in RPMI 1640. In the IL-2 superimposed TCR stimulation group, anti-CD3 mAb (1 μg/ml, eBioscience, CA, United States) and anti-CD28 mAb (1 μg/ml, eBioscience) were added at the same time as IL-2. After 24, 48, 72, and 96 h, respectively, the stimulated CD4^+^ T cells were seeded into 96-well plates. Then, 10 μl of Cell Counting Kit-8 (CCK-8) reagent was added to each well and incubated at 37°C in the presence of 5% CO_2_ for 1 h in the incubator. Absorbance was measured at 450 nm on a microplate reader.

### RNA Isolation and Real-Time PCR

Total RNA was isolated from CD4^+^ T cells with TRIzol Reagent (Invitrogen, CA, United States). Complementary DNA (cDNA) was synthesized from 1 μg total RNA with random primers and SuperScript II reverse transcriptase (Invitrogen, CA, United States), according to the manufacturer’s instructions. Briefly, the mixture of random primers, total RNA, and dNTP was heated at 65°C for 5 min and then placed on ice rapidly. First-strand buffer and DTT were added to the mixture and incubated at 25°C for 2 min. SuperScript II RT was added to the mixture and incubated for 10 min at 25°C, followed by 50 min at 42°C, and 15 min at 70°C. Real-time PCR with SYBR Green Master Mix (Thermo Fisher Scientific) was performed in triplicate on an ABI Prism 7500 (Thermo Fisher Scientific). After an initial denaturation step (2 min at 94°C), 45 cycles of PCR (15 s at 94°C, 30 s at 60°C) are performed. Human glyceraldehyde-3-phosphate dehydrogenase (GAPDH) was used as an endogenous control for data normalization. The 2^–ΔΔCt^ method was employed for analysis based on the following formula: ΔΔCt = (Ct_target gene_ − Ct_internal control_)_sample_ − (Ct_target gene_ − Ct_internal control_)_control_. The primers are listed in Table 2.

**TABLE 2:** Primer sequences for real-time qPCR.

	Forward primer	Reverse primer
p53	CTGCTCAGATAGCGATGGTC	TGTAGTTGTAGTGGATGGTGGTAC
CTCF	GAGGCTGCTGTGGACGAT	CAGGCAAAGGTAGGGTGTG
Foxp3	GAGAAGCTGAGTGCCATGCA	AGAGCCCTTGTCGGATGAT
IL10	TGAGAACAGCTGCACCCACTT	TCGGAGATCTCGAAGCATGTTA
RORγt	GCTGGTTAGGATGTGCCG	GGATGCTTTGGCGATGA
IL17A	CAATCCCACGAAATCCAGGATG	GGTGGAGATTCCAAGGTGAGG
GAPDH	AAGAGCTACGAGCTGCCTGAC	ATGGCCCAGCGGATGAG

### Western Blotting

CD4^+^ T cells were lysed with RIPA Lysis Buffer (Thermo Fisher Scientific) containing proteinase inhibitors (Thermo Fisher Scientific). Lysates were cleared at 14,000 × *g* and 4°C for 15 min, and protein concentration was determined by the Bradford protein assay (Bio-Rad, CA, United States). Proteins were separated by 8% sodium dodecyl sulfate–polyacrylamide gel electrophoresis (SDS-PAGE), and electrophoretically transferred onto polyvinylidene difluoride (PVDF) membranes (Bio-Rad, CA, United States). The membranes were then blocked with 5% bovine serum albumin (BSA) for 1 h at room temperature, washed twice with Tris-buffered saline containing Tween-20 (TBST), and separately incubated overnight at 4°C with primary antibodies, including mouse anti-p53 (1:1000 dilution), rabbit anti-CTCF (1:1000 dilution), rabbit anti-p300 (1:1000 dilution), and rabbit anti-GAPDH (1:1000 dilution) antibodies (Cell Signaling Technology, MA, United States). The membranes were washed with TBST and incubated with horseradish peroxidase-conjugated secondary antibodies, including horse anti-mouse (1:2000 dilution), goat anti-rabbit (1:2000 dilution) antibodies (Cell Signaling Technology) for 2 h at room temperature. Detection was performed with enhanced chemiluminescence (ECL) (Cell Signaling Technology) and relevant blots were quantified by densitometry by using the Quantity One software (Bio-Rad, CA, United States).

### Culture, and Transfection of Jurkat Cells

Jurkat cells were cultured in RPMI 1640 supplemented with 10% FBS and 1% penicillin/streptomycin and incubated at 37°C in presence of 5% CO_2_. The plasmids were transfected into Jurkat cells via Gene Pulser Xcell electroporation system (Bio-Rad). The transfection efficiency can reach about 60–70%.

### Chromatin Immunoprecipitation

Chromatin immunoprecipitation (ChIP) analysis was performed according to the instructions included in SimpleChIP^®^ Plus Sonication Chromatin IP Kit (Cell Signaling Technology, MA, United States). First, about 4 × 10^6^ CD4^+^ T cells were incubated in a medium containing 1% formaldehyde at room temperature, and cross-linking was stopped with glycine at a final concentration of 0.125 M. Cells were collected after two washes with ice-cold PBS and were subsequently lysed. The pellets and resuspended lysates were sonicated to reduce DNA into 500–1000 base pair fragments. Then, anti-CTCF, anti-p300, anti-H3K9ac, anti-H3K14ac, or control rabbit IgG antibodies were added for overnight incubation. All antibodies were obtained from Cell Signaling Technology. Protein A agarose beads were added to collect protein-DNA complexes. Samples were then decross-linked overnight at 65°C with sodium chloride (final concentration, 0.2 M) after washing. Purified DNA was used to amplify the target fragment by PCR or real-time PCR. Primers used were as follows: Forward 1 (−1335 to −1313):5′CAGGGGAGTGGAGAGAGAAACT3′ and Reverse 1 (−1194 to −1218):5′AATAAAAATGCGGACTCTGAACTG3′, Forward 2 (−694 to −670):5′TCCCCCTACCGAGTCCCGCGG TA3′ and Reverse 2 (−515 to −536):5′CTATATCAGTGCTGGG TAGCA3′, Forward 3 (−15 to +3):5′AGCCTCGCAGGGGTTG AT3′ and Reverse 3 (+130 to +112):5′GCCCGAACGCAAAG TGTC3′.

### Co-immunoprecipitation

Cellular proteins were extracted with RIPA lysis buffer. Then, 2 μg anti-CTCF or anti-p300 antibodies were added and incubated overnight at 4°C. ChIP-Grade protein A/G PLUS-agarose beads (100 μl, Millipore) were added to each IP reaction and incubated for 2 h at 4°C with shaking. Agarose beads were harvested by centrifugation at 3000 × *g* for 2 min, removing the supernatant. The precipitated complex was washed three times. The proteins were then eluted from the solid support using SDS sample loading buffer. The complexes were analyzed by Western blotting with anti-CTCF and anti-p300 antibodies.

### Statistical Analysis

The data were analyzed by SPSS 22.0 software. Continuous variables with normal distribution were presents as mean ± standard deviation (SD); non-normal variables were reported as median (interquartile range). Means of two continuous normally distributed variables were compared by independent samples Student’s test (data from different transfections were compared by paired *t* test and other data were compared by 2-group *t* test). Mann–Whitney *U* test and Kruskal–Wallis test were used, respectively, to compare the means of two groups of variables not normally distributed. Correlations were analyzed using Pearson’s correlation coefficient. Significance was set as *P* ≤ 0.05.

## Results

### Expression Levels of p53 in CD4^+^ T Cells From aGVHD Cases and Patients Without aGVHD

**FIGURE 1:**
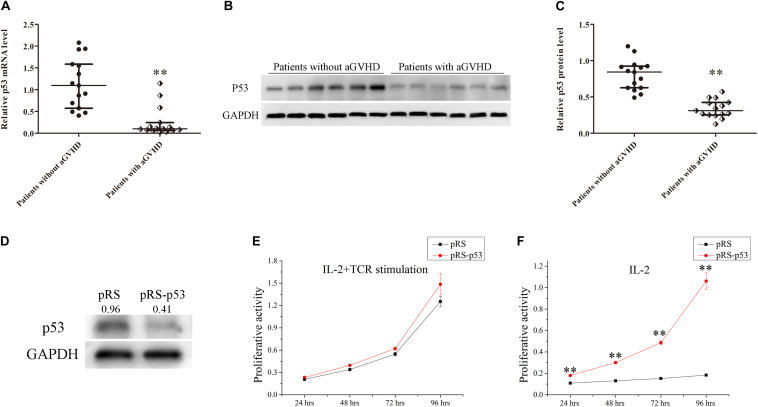
Expression levels of p53 in CD4^+^ T cells from patients with aGVHD and individuals without aGVHD. **(A)** Relative mRNA levels of p53 in CD4^+^ T cells from patients with aGVHD (*n* = 15) and the non-aGVHD group (*n* = 15) normalized to GAPDH expression. **(B)** Representative Western blot results for p53 protein expression in CD4^+^ T cells from patients with aGVHD and those without aGVHD. **(C)** Quantitative analysis of band intensities for p53 protein levels normalized to GAPDH expression (*n* = 15 per group). **(D)** Representative Western blotting results showing p53 protein levels in normal CD4^+^ T cells transfected with the p53 interference (pRS-p53) or negative control (pRS) plasmid. **(E,F)** Determination of the proliferation of p53-deficient CD4^+^ T cells after stimulation with IL-2 in the presence or absence of anti-CD3/CD28 antibodies. Data are mean ± SD from three independent experiments. (*^∗∗^P* < 0.01).

To investigate the relationship between p53 expression and aGVHD occurrence, the expression levels of p53 were detected in CD4^+^ T cells from patients with aGVHD and the non-aGVHD group. Real-time PCR and Western blot showed that p53 was significantly downregulated in CD4^+^ T cells from patients with aGVHD compared with the non-aGVHD group (Figures 1A–C).

In addition, to assess the effect of p53 on the proliferation of CD4^+^ T cells, the p53 interference (pRS-p53) or negative control (pRS) plasmid was transfected into CD4^+^ T cells from healthy donors. Then, the transfected cells were stimulated with IL-2 in the presence or absence of anti-CD3/CD28 antibodies (TCR stimulation). Western blot showed that p53 was significantly downregulated in pRS-p53 transfected cells compared with the pRS transfected cells (Figure 1D). Although the pRS-p53 and pRS groups had equivalent responses to IL-2 in the presence of anti-CD3/CD28 antibodies (Figure 1E), a striking difference was observed in the absence of TCR stimulation (Figure 1F). The pRS-p53 group proliferated strongly in response to IL-2 alone, at a magnitude comparable to their response to anti-CD3/anti-CD28 antibodies plus IL-2, whereas the pRS group exhibited a very limited expansion upon stimulation with IL-2 alone (Figures 1E,F). These results indicated that p53 downregulation in CD4^+^ T cells may lead to an over-reaction to IL-2 and aGVHD occurrence.

### CTCF Downregulation Reduces p53 Expression in CD4^+^ T Cells From Patients With aGVHD

**FIGURE 2:**
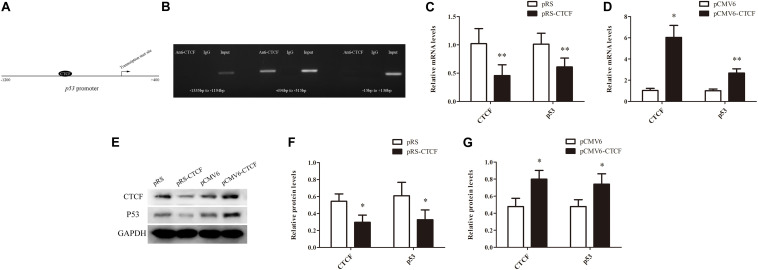
CTCF is a key factor to promote p53 expression in CD4^+^ T cells. **(A)** Schematic diagram of the CTCF binding site in the p53 promoter region. **(B)** ChIP-PCR showing that CTCF binds to the promoter region (−694 to −515) of p53 in T cells. **(C**,**D)** p53 mRNA levels in normal CD4^+^ T cells after CTCF silencing or overexpression. Data are mean ± SD from three independent experiments. **(E)** Representative Western blotting results for p53 protein expression in CD4^+^ T cells after CTCF silencing or overexpression. **(F**,**G)** Quantitative analysis of band intensities for p53 protein, normalized to GAPDH expression. Data are mean ± SD from three independent experiments. (*^∗^P* < 0.05, *^∗∗^P* < 0.01).

In order to explore the molecular mechanism underpinning p53 downregulation in CD4^+^ T cells from aGVHD patients, the promoter sequence of *p53* was analyzed with an online software^1^. The results showed that this region contains the CTCF binding site (Figure 2A). First, whether CTCF could bind to the *p53* promoter was explored by ChIP-PCR analysis in CTCF overexpressing Jurkat cells. Three pairs of primers covering the *p53* promoter region (−1335 to +130 bp) were evaluated. The results revealed that CTCF could indeed bind to the *p53* promoter at −694 bp to −515 bp (Figure 2B). Further, to investigate the effect of CTCF on p53 expression in CD4^+^ T cells, the CTCF interference (pRS-CTCF) and CTCF overexpression (pCMV6-CTCF) plasmids were transfected, respectively, into normal CD4^+^ T cells. The results showed that p53 was significantly downregulated in normal CD4^+^ T cells after CTCF silencing (Figures 2C,E,F). Conversely, p53 was significantly upregulated in normal CD4^+^ T cells after CTCF overexpression (Figures 2D,E,G).

**FIGURE 3:**
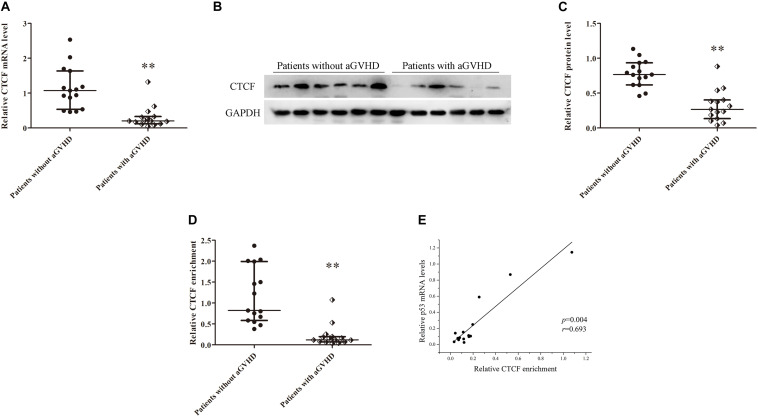
CTCF downregulation results in low expression of p53 in CD4^+^ T cells from patients with aGVHD. **(A)** Relative mRNA levels of CTCF in CD4^+^ T cells from patients with aGVHD (*n* = 15) and those without aGVHD (*n* = 15), normalized to GAPDH. **(B)** Representative Western blotting results for CTCF protein expression in CD4^+^ T cells from patients with aGVHD (*n* = 10) and those without aGVHD (*n* = 10). **(C)** Quantitative analysis of band intensities for CTCF protein, normalized to GAPDH expression. **(D)** ChIP-qPCR analysis of CTCF enrichment in the p53 promoter in chromatin fractions extracted from CD4^+^ T cells from patients with aGVHD (*n* = 15) and individuals without aGVHD (*n* = 15). Values are relative to those obtained with input DNA prepared from untreated chromatin. **(E)** Correlation between CTCF enrichment and p53 mRNA levels in CD4^+^ T cells from aGVHD cases (*n* = 15). (*^∗∗^P* < 0.01).

In addition, CTCF expression levels were assessed in CD4^+^ T cells from patients with aGVHD and those without. CCCTC-binding factor amounts in CD4^+^ T cells from patients with aGVHD were significantly lower than those of the non-aGVHD group (Figures 3A–C). Meanwhile, CTCF binding to the *p53* promoter region was compared between aGVHD patients and individuals without aGVHD by ChIP-qPCR with anti-CTCF antibodies. As shown in Figure 3D, aGVHD patients had reduced binding of CTCF to the *p53* promoter compared with the non-aGVHD group. To investigate the relationship between CTCF binding to the *p53* promoter and p53 expression in CD4^+^ T cells from aGVHD cases, correlation analysis was performed. The results indicated that the CTCF binding level was positively correlated with p53 expression in CD4^+^ T cells from aGVHD patients (Figure 3E). Taken together, the above findings suggested that CTCF is a key factor promoting p53 expression, and decreased CTCF expression might be one of the main causes of p53 downregulation in CD4^+^ T cells from aGVHD cases.

### CTCF Recruits p300 to Bind to the *p53* Promoter

**FIGURE 4:**
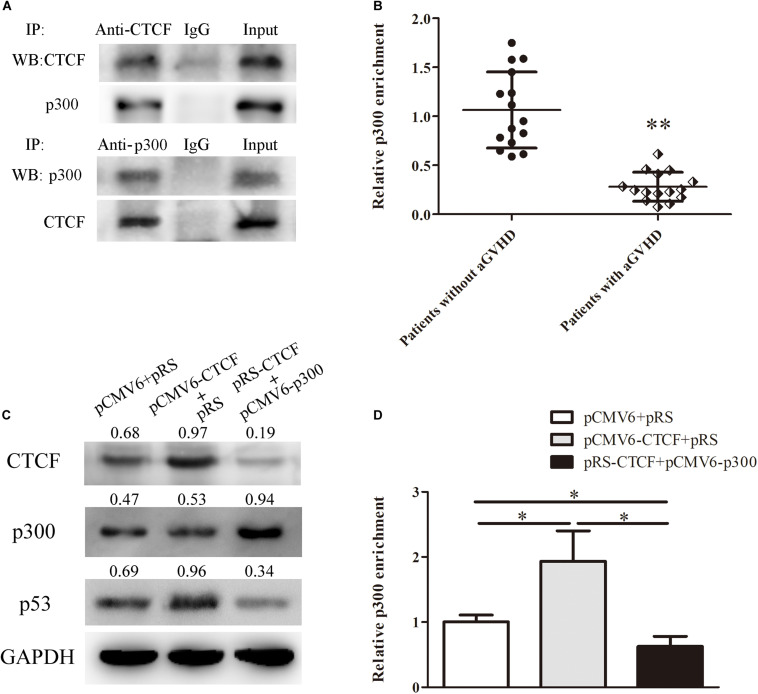
CTCF recruits p300 for interaction with the *p53* promoter. **(A)** Co-immunoprecipitation with anti-CTCF (upper panel) or anti-p300 (lower panel) antibodies in Jurkat cells after CTCF and p300 overexpression; detection of the CTCF and p300 complex was performed by Western blotting. **(B)** ChIP-qPCR analysis of p300 enrichment in the p53 promoter in chromatin fractions extracted from CD4^+^ T cells from patients with aGVHD (*n* = 15) and individuals without aGVHD (*n* = 15). Values are relative to those obtained with input DNA prepared from untreated chromatin. **(C)** Representative Western blotting results for p53 protein expression in CD4^+^ T cells after CTCF or p300 overexpression and CTCF silencing. Data are mean ± SD from three independent experiments. **(D)** ChIP-qPCR analysis of p300 enrichment in the p53 promoter in chromatin fractions extracted from CD4^+^ T cells after CTCF or p300 overexpression and CTCF knockdown. Values are relative to those obtained with input DNA prepared from untreated chromatin. Data are mean ± SD from three independent experiments. ^∗^*P* < 0.05, *^∗∗^P* < 0.01.

In order to unveil the molecular mechanism by which CTCF promotes p53 expression, the STRING database^2^ was used to predict proteins that might interact with CTCF. In this study, p300, a histone acetylase, was predicted to interact with CTCF. First, to confirm the interaction between CTCF and p300, CTCF and p300 expression plasmids were transfected into Jurkat cells and whether CTCF could form a complex with p300 was assessed by co-immunoprecipitation. As shown in Figure 4A, CTCF indeed co-precipitated with p300. Further, the binding levels of p300 in the promoter region of *p53* were determined by ChIP-qPCR in CD4^+^ T cells from patients with aGVHD and the non-aGVHD group. As shown in Figure 4B, patients with aGVHD showed reduced binding of p300 to the *p53* promoter compared with the non-aGVHD group.

Next, to investigate the role of CTCF in p300 binding to the *p53* promoter, the CTCF overexpression, p300 overexpression, and CTCF interference plasmids were transfected into normal CD4^+^ T cells. Compared with the negative control group, p300 binding to the *p53* promoter and p53 expression levels were increased in the CTCF overexpression group (Figures 4C,D). However, after the p300 overexpression and CTCF interference plasmids were co-transfected into normal CD4^+^ T cells, p300 binding and p53 expression levels were significantly lower compared with the CTCF overexpression and negative control groups (Figures 4C,D). These data strongly suggested that CTCF plays a crucial role in recruiting p300 to the *p53* promoter.

### CTCF Modifies Histones H3K9ac and H3K14ac in the *p53* Promoter

**FIGURE 5:**
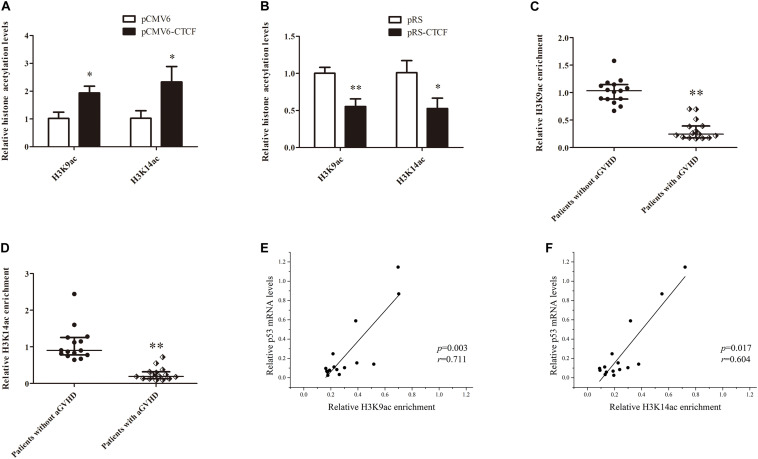
CTCF alters histone H3K9ac and H3K14ac levels in the *p53* promoter. **(A,B)** ChIP-qPCR analysis of the enrichment of histone H3K9ac and H3K14ac levels in the *p53* promoter region in normal CD4^+^ T cells transfected with pCMV6-CTCF **(A)** or pRS-CTCF **(B)**. Values are relative to those obtained with input DNA prepared from untreated chromatin. Data are mean ± SD from three independent experiments. **(C,D)** ChIP-qPCR analysis of the enrichment of histone H3K9ac **(C)** and H3K14ac **(D)** levels in the p53 promoter region in CD4^+^ T cells from patients with aGVHD (*n* = 15) and individuals without aGVHD (*n* = 15). Values are relative to those obtained with input DNA prepared from untreated chromatin. **(E**,**F)** Correlation analysis of H3K9ac **(E)**/H3K14ac **(F)** of *p53* promoter and p53 expression in aGVHD CD4^+^ T cells (*n* = 15).*^∗^P* < 0.05, *^∗∗^P* < 0.01.

Histone H3K9ac and H3K14ac are critical epigenetic modifications for upregulated gene transcription. Meanwhile, p300 is a key acetylase that mediates the acetylation of both H3K9 and H3K14. To confirm the effects of CTCF on H3K9ac and H3K14ac in the *p53* promoter region, H3K9ac and H3K14ac levels in the *p53* promoter region were detected in normal CD4^+^ T cells transfected with CTCF overexpression or interference plasmid by ChIP-qPCR. The results showed increased histone H3K9ac and H3K14ac levels in the *p53* promoter region after ectopic expression of CTCF in normal CD4^+^ T cells (Figure 5A). Conversely, H3K9ac and H3K14ac were significantly downregulated in normal CD4^+^ T cells after CTCF interference (Figure 5B). Further, H3K9ac and H3K14ac in the *p53* promoter region were assessed in CD4^+^ T cells from patients with aGVHD and the non-aGVHD group. ChIP-qPCR showed that the levels of H3K9ac and H3K14ac in the *p53* promoter region in patients with aGVHD were significantly lower than those of controls (Figures 5C,D). In addition, correlation analysis of H3K9/K14ac of *p53* promoter and p53 expression were performed. We found that H3K9/K14ac were positively correlated with p53 expression in aGVHD CD4^+^ T cells (Figures 5E,F). Taken together, these results indicated that CTCF promotes p53 expression by increasing H3K9ac and H3K14ac levels in the *p53* promoter region in CD4^+^ T cells.

### Restoring CTCF Expression in CD4^+^ T Cells From aGVHD Cases Improves Treg/Th17 Cell Imbalance by Upregulating p53

**FIGURE 6:**
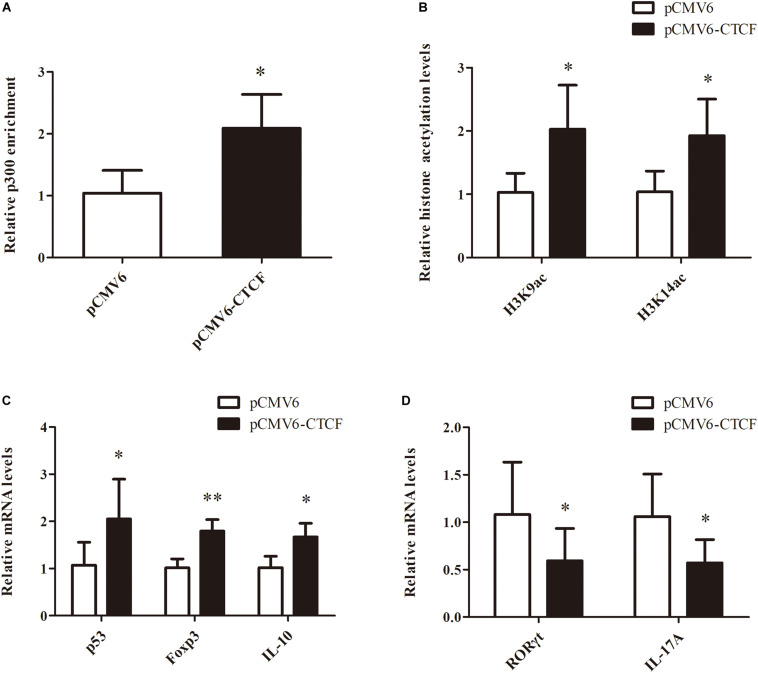
Restoring CTCF expression in CD4^+^ T cells from aGVHD cases improves Treg/Th17 cell imbalance by increasing p53 expression. **(A**,**B)** ChIP-qPCR analysis of the enrichment of p300 binding **(A)** and H3K9/K14 **(B)** acetylation levels in the *p53* promoter region in CD4^+^ T cells from three separate aGVHD cases after CTCF overexpression. Values are relative to those obtained with input DNA prepared from untreated chromatin. Data are mean ± SD from three independent experiments. **(C**,**D)** Real-time PCR analysis of p53, Foxp3, IL-10 **(C)**, RORγt, and IL-17A **(D)** expression levels in CD4^+^ T cells from three separate aGVHD cases after CTCF overexpression. Data are mean ± SD from three independent experiments. *^∗^P* < 0.05, *^∗∗^P* < 0.01.

To determine the effects of CTCF upregulation in CD4^+^ T cells from aGVHD cases on Treg/Th17 cell imbalance, p300 binding and H3K9/K14 acetylation levels in the p53 promoter region were assessed, as well as p53, Foxp3, IL-10, RORγt, and IL-17A levels, after overexpression of CTCF in CD4^+^ T cells from aGVHD cases. As expected, significantly increased p300 binding and H3K9/K14 acetylation levels in the *p53* promoter region were observed in CD4^+^ T cells from aGVHD patients after CTCF overexpression (Figures 6A,B). Similarly, p53, Foxp3, and IL-10 expression levels were increased (Figure 6C), while RORγt and IL-17A were downregulated in CTCF-overexpressing CD4^+^ T cells from aGVHD patients (Figure 6D). Taken together, these results suggested that CTCF upregulation in CD4^+^ T cells from aGVHD cases could improve the Treg/Th17 cell imbalance by increasing p53 expression.

## Discussion

This study demonstrated that p53 downregulation significantly enhanced CD4^+^ T cell response to IL-2 stimulation. Normal CD4^+^ T cells, with intact p53, failed to proliferate in response to IL-2 in the absence of TCR activation. However, after p53 silencing in normal CD4^+^ T cells, stimulation with IL-2 alone could lead to the proliferation of CD4^+^ T cells. These findings suggest that the TCR dependence of CD4^+^ T cell response to IL-2 stimulation is mediated by p53. Other studies have found that p53 expression can be increased significantly and continuously in CD4^+^ T cells stimulated with IL-2 alone. In contrast, stimulation of antigen-presenting cells with IL-2 induces only transient p53 protein upregulation, terminated by a significant downregulation to the pre-stimulation baseline (25). These findings suggest that sustained expression of p53 blocks the response of CD4^+^ T cells to IL-2, while TCR activation overcomes this obstacle by reducing p53 expression. Therefore, constant p53 expression plays an important role in maintaining the stability of CD4^+^ T cells and preventing excessive activation and proliferation. Loss of p53 expression may lead to overactivation of CD4^+^ T cells, inducing inflammatory and/or autoimmune diseases. CD4^+^ T cell-specific p53-deficient mice spontaneously develop inflammatory lesions in various organs, including the lung, liver, stomach, thyroid, submandibular gland, and kidney (15). Currently, whether p53 expression is defective in CD4^+^ T cells of aGVHD patients and whether excessive activation of CD4^+^ T cells in these patients is related to abnormal p53 expression remain unclear. The present study showed that p53 was significantly downregulated in CD4^+^ T cells from patients with aGVHD compared with the non-aGVHD group. Moreover, other studies have confirmed that IL-2 is significantly increased in both aGVHD mouse models and patients (26). These findings suggest that CD4^+^ T cells with insufficient p53 expression are over-activated and proliferate under stimulation by large amounts of IL-2, mediating inflammatory damage in various organs in aGVHD patients.

It is known that TCR signal downregulates p53 expression (15), so it can be well predicted that activated CD4^+^ T cells express lower p53 levels, and different Th cell subsets may also express different levels of p53. H. Kawashima et al. found a decreased Foxp3 expression and an increased RORγt expression in CD4^+^ T cells of p53 knockout mice compared with wild-type mice. There is no significant change in the expression of Th1 type transcription factor T-bet and Th2 type transcription factor Gata3 (15). These results suggest that p53 may mainly affect the differentiation of Treg and Th17, but not Th1/Th2. At the beginning of this study, we intended to explore the relationship between the decreased p53 expression and the activated status or components of Th subsets in CD4^+^ T cells from aGVHD patients. However, the number of CD4^+^ T cells isolated from patients with aGVHD is small, so it is difficult to further separate the subsets of Th1, Th2, Treg, and Th17 cells for subsequent detection. In the follow-up study, we will continue to explore relevant issues by building aGVHD animal models.

CCCTC-binding factor is a multifunctional transcription factor with multiple zinc finger structures that binds to the DNA and regulates gene expression through a variety of mechanisms. It is essential and highly conserved from *Drosophila* to mice and humans (27, 28). CCCTC-binding factor is involved in transcriptional regulation by binding to chromatin insulators and preventing interactions between promoters and nearby enhancers and silencers. It acts as a transcriptional repressor by binding to the promoters of the MYC proto-oncogene (*c-Myc*), BCL2-associated X protein (*BAX*), and homeobox A10 (*HOXA10*) (29–31). However, CTCF can bind to the amyloid beta precursor protein (*APP*) promoter as a transcriptional activator, upregulating APP (32). In recent years, many studies have found that CTCF may be closely related to autoimmune diseases. CCCTC-binding factor can mediate DNA hydroxymethylation and can contribute to overexpression of suppressor of cytokine signaling 1 (SOCS1) in systemic lupus erythematosus (SLE) CD4^+^ T cells through binding to the promoter region of SOCS1 (33). CCCTC-binding factor regulates histone modifications in the MHC class II (MHC-II) genes *HLA-DRB1* and *HLA-DQA1* promoter regions, inducing the transcription of HLA-DRB1 and HLA-DQA1 (34). CCCTC-binding factor interacts with POU class 2 homeobox 1 (Oct-1) to directly bind to the *IL-17* promoter region, downregulating IL-17 and preventing the differentiation of Th17 cells (35). However, the relationship between CTCF and aGVHD remains undefined. In this study, CTCF recruited p300 to the *p53* promoter region and induced p53 expression by increasing histone H3K9/K14 acetylation levels in this region. CCCTC-binding factor expression levels in CD4^+^ T cells from aGVHD cases were significantly reduced, which further decreased p53 expression in these cells. In HeLa cells, CTCF promotes p53 transcription by preventing the formation of repressive histone markers such as H3K9/K27 and H4K20 methylation in the *p53* promoter region (36). This study unveiled the molecular mechanism of p53 downregulation in CD4^+^ T cells from aGVHD cases and further explained the epigenetic mechanism by which CTCF regulates p53 expression.

In addition, Co-IP requires a relatively large amount of cells, so we chose to use cell lines for this experiment. There is an interaction between CTCF and p300 in primary human CD4^+^ T cells because the binding level of P300 in p53 promoter was significantly increased after overexpression of CTCF, while the binding level of P300 in p53 promoter was significantly decreased after CTCF interference, which reflects that CTCF is an important protein that mediates P300 binding to p53 promoter. From the current study, CTCF should have an indirect effect on the regulation of p53 expression. We have confirmed that CTCF regulates the p53 expression through p300 affecting H3K9/K14 acetylation. In addition, there may be some other mechanisms, such as histone methylation and DNA methylation, etc. Our follow-up work will continue to explore these mechanisms.

Accumulating evidence suggests that the imbalance between Th17 cells and Tregs plays a critical role in aGVHD (17–19, 37–39). Our previous research revealed that STAT3 overexpression and activation in CD4^+^ T cells from aGVHD cases is one of the causes of Th17/Treg imbalance (6, 22). In this study, after CTCF overexpression in CD4^+^ T cells from aGVHD patients, p53, Foxp3, and IL-10 expression levels were significantly increased, while RORγt and IL-17A were significantly downregulated, thus reversing the Th17/Treg imbalance. It is known that p53 downregulates Th17-related genes and upregulates Treg-associated genes (14, 15). After CTCF overexpression, p53 expression was restored and the imbalance of Th17/Treg was corrected. In addition, CTCF directly binds to the IL-17A promoter and inhibits its expression (35). Therefore, CTCF overexpression in CD4^+^ T cells from aGVHD cases effectively reverses the Th17/Treg imbalance.

In summary, this study demonstrated that low levels of histone H3K9/K14 acetylation in the *p53* promoter region, caused by reduced CTCF expression, constitute an important cause of p53 downregulation in CD4^+^ T cells from aGVHD cases. Insufficient p53 expression leads to excessive activation of CD4^+^ T cells and an imbalance of Th17/Treg, which in turn mediate the occurrence of inflammatory damage to various organs in aGVHD patients. This study clarified the molecular mechanism of the low expression of p53 in aGVHD CD4^+^ T cells, further improved the cause of aGVHD, and provided a reference target for the effective prevention and treatment of aGVHD.

## Data Availability Statement

All datasets presented in this study are included in the article/supplementary material.

## Ethics Statement

The study protocol was approved by the Human Ethics Committee of Xiangya School of Medicine, Central South University. All subjects provided signed informed consent in accordance with the Declaration of Helsinki.

## Author Contributions

YX, JH, YC, and BF contributed to the design and planning of the experiments. XC, XX, and JH provided samples. XC, XX, S-HY, CC and JH conducted the laboratory experimental work. JH, YC, BF, and YX contributed to the reporting of findings and writing of the manuscript. All authors critically revised the manuscript and gave final approval of the version to be submitted.

## Conflict of Interest

The authors declare that the research was conducted in the absence of any commercial or financial relationships that could be construed as a potential conflict of interest.

## Footnotes

1 http://jaspar.binf.ku.dk/

2 https://string-db.org/cgi/input.pl

## References

[1] Martin PJ Rizzo JD Wingard JR Ballen K Curtin PT Cutler C First- and second-line systemic treatment of acute graft-versus-host disease: recommendations of the American Society of Blood and Marrow Transplantation. *Biol Blood Marr Transplant.* (2012) 18:1150–63. 10.1016/j.bbmt.2012.04.005 22510384 PMC3404151

[2] Wolff D Ayuk F Elmaagacli A Bertz H Lawitschka A Schleuning M Current practice in diagnosis and treatment of acute graft-versus-host disease: results from a survey among German-Austrian-Swiss hematopoietic stem cell transplant centers. *Biol Blood Marr Transplantat.* (2013) 19:767–76. 10.1016/j.bbmt.2013.01.018 23376495

[3] Chen Y Xu Y Fu G Liu Y Peng J Fu B Allogeneic hematopoietic stem cell transplantation for patients with acute leukemia. *Chinese J Cancer Res Chung Cheng Yen Chiu.* (2013) 25:389–96. 10.3978/j.issn.1000-9604.2013.07.01 PMC3752360 23997525

[4] Zeiser R Socie G Blazar BR. Pathogenesis of acute graft-versus-host disease: from intestinal microbiota alterations to donor T cell activation. *Br J Haematol.* (2016) 175:191–207. 10.1111/bjh.14295 27619472

[5] Zitzer NC Snyder K Meng X Taylor PA Efebera YA Devine SM MicroRNA-155 modulates acute graft-versus-host disease by impacting t cell expansion, migration, and effector function. *J Immunol.* (2018) 200:4170–9. 10.4049/jimmunol.1701465 29720426 PMC5988971

[6] Xu YJ Li L Chen Y Fu B Wu DS Li XL Role of HMGB1 in regulation of STAT3 expression in CD4(+) T cells from patients with aGVHD after allogeneic hematopoietic stem cell transplantation. *Clin Immunol.* (2015) 161:278–83. 10.1016/j.clim.2015.08.012 26327693

[7] Fierabracci A Pellegrino M. The Double Role of p53 in Cancer and Autoimmunity and Its Potential as Therapeutic Target. *Int J Mol Sci.* (2016) 17:1975. 10.3390/ijms17121975 27897991 PMC5187775

[8] Duffy MJ Synnott NC McGowan PM Crown J O’Connor D Gallagher WM. p53 as a target for the treatment of cancer. *Cancer Treat Rev.* (2014) 40:1153–60. 10.1016/j.ctrv.2014.10.004 25455730

[9] Mao C Wang X Liu Y Wang M Yan B Jiang Y A G3BP1-interacting lncRNA promotes ferroptosis and apoptosis in cancer via nuclear sequestration of p53. *Cancer Res.* (2018) 78:3484–96. 29588351 10.1158/0008-5472.CAN-17-3454 PMC8073197

[10] Takatori H Kawashima H Suzuki K Nakajima H. Role of p53 in systemic autoimmune diseases. *Crit Rev Immunol.* (2014) 34:509–16. 25597313 10.1615/critrevimmunol.2014012193

[11] Leech M Xue JR Dacumos A Hall P Santos L Yang Y The tumour suppressor gene p53 modulates the severity of antigen-induced arthritis and the systemic immune response. *Clin Exp Immunol.* (2008) 152:345–53. 10.1111/j.1365-2249.2008.03629.x 18341615 PMC2384110

[12] Simelyte E Rosengren S Boyle DL Corr M Green DR Firestein GS. Regulation of arthritis by p53: critical role of adaptive immunity. *Arthritis Rheum.* (2005) 52:1876–84. 10.1002/art.21099 15934085

[13] Munoz-Fontela C Mandinova A Aaronson SA Lee SW. Emerging roles of p53 and other tumour-suppressor genes in immune regulation. *Nat Rev Immunol.* (2016) 16:741–50. 10.1038/nri.2016.99 27667712 PMC5325695

[14] Park JS Lim MA Cho ML Ryu JG Moon YM Jhun JY p53 controls autoimmune arthritis via STAT-mediated regulation of the Th17 cell/Treg cell balance in mice. *Arthritis Rheum.* (2013) 65:949–59. 10.1002/art.37841 23280308

[15] Kawashima H Takatori H Suzuki K Iwata A Yokota M Suto A Tumor suppressor p53 inhibits systemic autoimmune diseases by inducing regulatory T cells. *J Immunol.* (2013) 191:3614–23. 10.4049/jimmunol.1300509 24006461

[16] Zhang S Zheng M Kibe R Huang Y Marrero L Warren S Trp53 negatively regulates autoimmunity via the STAT3-Th17 axis. *FASEB J.* (2011) 25:2387–98. 10.1096/fj.10-175299 21471252 PMC3114529

[17] Fulton LM Carlson MJ Coghill JM Ott LE West ML Panoskaltsis-Mortari A Attenuation of acute graft-versus-host disease in the absence of the transcription factor RORgammat. *J Immunol.* (2012) 189:1765–72. 10.4049/jimmunol.1200858 22778391 PMC3411855

[18] Hoffmann P Ermann J Edinger M Fathman CG Strober S. Donor-type CD4(+)CD25(+) regulatory T cells suppress lethal acute graft-versus-host disease after allogeneic bone marrow transplantation. *J Exp Med.* (2002) 196:389–99. 10.1084/jem.20020399 12163567 PMC2193938

[19] Liu Y Cai Y Dai L Chen G Ma X Wang Y The expression of Th17-associated cytokines in human acute graft-versus-host disease. *Biol Blood Marr Transplant.* (2013) 19:1421–9. 10.1016/j.bbmt.2013.06.013 23792271

[20] Gates LA Shi J Rohira AD Feng Q Zhu B Bedford MT Acetylation on histone H3 lysine 9 mediates a switch from transcription initiation to elongation. *J Biol Chem.* (2017) 292:14456–72. 10.1074/jbc.m117.802074 28717009 PMC5582839

[21] Karmodiya K Krebs AR Oulad-Abdelghani M Kimura H Tora L. H3K9 and H3K14 acetylation co-occur at many gene regulatory elements, while H3K14ac marks a subset of inactive inducible promoters in mouse embryonic stem cells. *BMC Genomics.* (2012) 13:424. 10.1186/1471-2164-13-424 22920947 PMC3473242

[22] Xu YJ Chen FP Chen Y Fu B Liu EY Zou L To induce acute graft-vs.-host disease after hematopoietic stem cell transplantation: lack of sirtuin-1 in CD4(+) T Cells. *Front Immunol.* (2018) 9:3078. 10.3389/fimmu.2018.03078 30622543 PMC6308326

[23] Glucksberg H Storb R Fefer A Buckner CD Neiman PE Clift RA Clinical manifestations of graft-versus-host disease in human recipients of marrow from HL-A-matched sibling donors. *Transplantation.* (1974) 18:295–304. 10.1097/00007890-197410000-00001 4153799

[24] Przepiorka D Weisdorf D Martin P Klingemann HG Beatty P Hows J 1994 consensus conference on acute GVHD grading. *Bone Marr Transplantat.* (1995) 15:825–8. 7581076

[25] Watanabe M Moon KD Vacchio MS Hathcock KS Hodes RJ. Downmodulation of tumor suppressor p53 by T cell receptor signaling is critical for antigen-specific CD4(+) T cell responses. *Immunity.* (2014) 40:681–91. 10.1016/j.immuni.2014.04.006 24792911 PMC4073799

[26] Li X Chen T Gao Q Zhang W Xiao Y Zhu W A panel of 4 biomarkers for the early diagnosis and therapeutic efficacy of aGVHD. *JCI Insight.* (2019) 4:e130413. 10.1172/jci.insight.130413 31434801 PMC6777816

[27] Xiao T Wongtrakoongate P Trainor C Felsenfeld G. CTCF recruits centromeric protein CENP-E to the pericentromeric/centromeric regions of chromosomes through unusual CTCF-binding sites. *Cell Rep.* (2015) 12:1704–14. 10.1016/j.celrep.2015.08.005 26321640 PMC4633288

[28] Hou C Zhao H Tanimoto K Dean A. CTCF-dependent enhancer-blocking by alternative chromatin loop formation. *Proc Natl Acad Sci USA.* (2008) 105:20398–403. 10.1073/pnas.0808506106 19074263 PMC2629272

[29] Schuijers J Manteiga JC Weintraub AS Day DS Zamudio AV Hnisz D Transcriptional dysregulation of MYC reveals common enhancer-docking mechanism. *Cell Rep.* (2018) 23:349–60. 10.1016/j.celrep.2018.03.056 29641996 PMC5929158

[30] Mendez-Catala CF Gretton S Vostrov A Pugacheva E Farrar D Ito Y A novel mechanism for CTCF in the epigenetic regulation of Bax in breast cancer cells. *Neoplasia.* (2013) 15:898–912. 10.1593/neo.121948 23908591 PMC3730042

[31] Mustafa M Lee JY Kim MH. CTCF negatively regulates HOXA10 expression in breast cancer cells. *Biochem Biophys Res Commun.* (2015) 467:828–34. 10.1016/j.bbrc.2015.10.058 26478432

[32] Vostrov AA Taheny MJ Quitschke WW. A region to the N-terminal side of the CTCF zinc finger domain is essential for activating transcription from the amyloid precursor protein promoter. *J Biol Chem.* (2002) 277:1619–27. 10.1074/jbc.M109748200 11706010

[33] Zhao M Wang J Liao W Li D Li M Wu H Increased 5-hydroxymethylcytosine in CD4(+) T cells in systemic lupus erythematosus. *J Autoimmun.* (2016) 69:64–73. 10.1016/j.jaut.2016.03.001 26984631

[34] Majumder P Gomez JA Chadwick BP Boss JM. The insulator factor CTCF controls MHC class II gene expression and is required for the formation of long-distance chromatin interactions. *J Exp Med.* (2008) 205:785–98. 10.1084/jem.20071843 18347100 PMC2292219

[35] Kim LK Esplugues E Zorca CE Parisi F Kluger Y Kim TH Oct-1 regulates IL-17 expression by directing interchromosomal associations in conjunction with CTCF in T cells. *Mol Cell.* (2014) 54:56–66. 10.1016/j.molcel.2014.02.004 24613343 PMC4058095

[36] Soto-Reyes E Recillas-Targa F. Epigenetic regulation of the human p53 gene promoter by the CTCF transcription factor in transformed cell lines. *Oncogene.* (2010) 29:2217–27. 10.1038/onc.2009.509 20101205

[37] Carlson MJ West ML Coghill JM Panoskaltsis-Mortari A Blazar BR Serody JS. In vitro-differentiated TH17 cells mediate lethal acute graft-versus-host disease with severe cutaneous and pulmonary pathologic manifestations. *Blood.* (2009) 113:1365–74. 10.1182/blood-2008-06-162420 18957685 PMC2637199

[38] Cohen JL Trenado A Vasey D Klatzmann D Salomon BL. CD4(+)CD25(+) immunoregulatory T cells: new therapeutics for graft-versus-host disease. *J Exp Med.* (2002) 196:401–6. 10.1084/jem.20020090 12163568 PMC2193933

[39] Kingsley CI Karim M Bushell AR Wood KJ. CD25+CD4+ regulatory T cells prevent graft rejection: CTLA-4- and IL-10-dependent immunoregulation of alloresponses. *J Immunol.* (2002) 168:1080–6. 10.4049/jimmunol.168.3.1080 11801641

